# A Meta-Analysis of Stressors from the Total Environment Associated with Children’s General Cognitive Ability

**DOI:** 10.3390/ijerph17155451

**Published:** 2020-07-29

**Authors:** Frances M. Nilsen, Jazmin D.C. Ruiz, Nicolle S. Tulve

**Affiliations:** 1 Office of Research and Development, U.S. Environmental Protection Agency Research Triangle Park, Durham, NC 27709, USA; jazmin.ruiz@honeywell.com (J.D.C.R.); tulve.nicolle@epa.gov (N.S.T.); 2 Honeywell International, Buffalo, NY 14210, USA

**Keywords:** children, cognition, development, environmental health, chemical, non-chemical, risk factors

## Abstract

General cognitive ability, often referred to as ‘general intelligence’, comprises a variety of correlated abilities. Childhood general cognitive ability is a well-studied area of research and can be used to predict social outcomes and perceived success. Early life stage (e.g., prenatal, postnatal, toddler) exposures to stressors (i.e., chemical and non-chemical stressors from the total (built, natural, social) environment) can impact the development of childhood cognitive ability. Building from our systematic scoping review (Ruiz et al., 2016), we conducted a meta-analysis to evaluate more than 100 stressors related to cognitive development. Our meta-analysis identified 23 stressors with a significant increase in their likelihood to influence childhood cognitive ability by 10% or more, and 80 stressors were observed to have a statistically significant effect on cognitive ability. Stressors most impactful to cognition during the prenatal period were related to maternal health and the mother’s ability to access information relevant to a healthy pregnancy (e.g., diet, lifestyle). Stressors most impactful to cognition during the early childhood period were dietary nutrients (infancy), quality of social interaction (toddler), and exposure to toxic substances (throughout early childhood). In conducting this analysis, we examined the relative impact of real-world exposures on cognitive development to attempt to understand the inter-relationships between exposures to both chemical and non-chemical stressors and early developmental life stages. Our findings suggest that the stressors observed to be the most influential to childhood cognitive ability are not permanent and can be broadly categorized as activities/behaviors which can be modified to improve childhood cognition. This meta-analysis supports the idea that there are complex relationships between a child’s total environment and early cognitive development.

## 1. Introduction

General cognitive ability, often referred to as ‘general intelligence’, comprises a variety of correlated abilities including spatial and verbal abilities, information processing speed, and memory [1,2]. Childhood general cognitive ability is a well-studied area of research and can be used to predict social outcomes and perceived success [2,3]. As such, the World Health Organization (WHO) reports that conditions that allow cognitive ability to flourish are vital to children’s lifelong success. The field of cognitive development is moving towards a more comprehensive framework for characterizing cognition [4]. Recent studies have shown the importance of both child-specific inherent characteristics and environmental factors, as well as the interactions between factors related to early childhood cognitive development [5]. However, research generally emphasizes the child or the environment, and the peripheral factors that influence cognitive ability are seldom taken into account [4,5,6,7,8,9,10]. The complex inter-relationships between chemical and non-chemical stressors and their possible collective/synergistic/antagonistic effect(s) on cognitive development have yet to be fully elucidated.

Early life stage (e.g., prenatal, postnatal, toddler) exposures to chemical and non-chemical stressors from the total (built, natural, social) environment can impact the development of childhood cognitive ability [1,11,12,13,14]. While identifying multiple chemical and non-chemical stressors from the home, community, and social environments is a methodological challenge, many studies have examined specific groups of stressors [15,16,17,18]. Exposure to certain chemicals during critical windows of development causes neurotoxicity through alterations in brain structure and function and may lead to diminished cognitive development [17,18,19,20,21,22,23,24,25,26]. Exposures to social stressors, particularly those related to socioeconomics, have been shown to affect childhood cognitive ability in a variety of cohorts [7,27,28,29,30,31]. Activities, such as diet and sleep patterns, have been shown to be correlated to cognitive outcomes in children [32,33,34,35,36,37]. Stressors experienced during early life stages that coincide with sensorimotor development (infancy) and pre-operational development (toddler and early childhood) could affect school-age readiness and cognitive performance into adolescence when inherent factors (i.e., genetics) gain increasing influence on cognitive ability [38,39,40,41,42,43,44,45,46,47].

In a systematic scoping review, Ruiz et al. [48] evaluated more than 100 stressors related to cognitive development, including inherent characteristics, and activities and behaviors. To better understand how development of cognitive ability is affected by the interactions of multiple stressors, Ruiz et al. [48] probed the links between chemical and non-chemical stressors, inherent characteristics, and activities and behaviors. Ruiz et al. concluded that: (1) positive impacts to cognition were largely attributable to non-chemical stressors related to the mother and child; (2) negative impacts were attributed to both chemical and non-chemical stressors in the child’s total environment; and (3) the true etiology of cognitive ability is likely a combination of stressors experienced throughout a child’s life course [48]. To date, this review is the most comprehensive analysis of the interactions of chemical and non-chemical stressors from the total environment on childhood cognitive development. Eleven studies published since 2016 have used Ruiz et al. [48] as a springboard to examine the effects of specific stressors such as neighborhood chaos, industrial pollution, environmental contaminants, socioeconomic status, and poverty on childhood cognitive development [6,7,28,49,50,51,52,53,54,55,56]. Recent publications related to childhood cognitive development that do not cite Ruiz et al. [48] focus on the effects of postnatal care, maternal mental health, and physical brain characteristics [57,58,59,60].

Our objective was to identify key stressors by conducting a meta-analysis on those examined in the Ruiz et al. [48] systematic scoping review. The children’s health and well-being conceptual framework put forth by Tulve et al. [17] was used to organize the stressors into life stage-specific categories and discuss the impacts of each life stage and stressor combination on childhood cognitive ability. In doing this meta-analysis, we aimed to understand the effects of real-world exposures on cognitive development as opposed to their effects in a controlled setting such as randomized control and intervention studies.

## 2. Methods

### 2.1. Data Collection and Extraction

Ruiz et al. [48] identified more than 100 factors that influenced a child’s cognitive development and described the complex relationships that exist between those factors. Ruiz et al. [48] conducted a search of the peer-reviewed literature in PubMed, Web of Science, and PsycINFO databases using a combination of words associated with cognitive health (e.g., neurodevelopment or cognitive development) OR ((cognition or learning) AND association) AND children. Included studies were published in English and conducted in children from birth to 18 years of age.

#### 2.1.1. Inclusion Criteria

The inclusion criteria from Ruiz et al. [48] are as follows:

- Observational studies, randomized control trials, review or meta-analysis;
- Time of exposure to a determinant or stressor occurred at or before health outcome was assessed;
- Health outcome was measured in children under 18 years old;
- General cognitive outcome was measured using current and earlier versions of evidence-based assessments of cognitive functioning classified as well established [19] and expressed as a continuous variable or categorized as below average or significant cognitive delay (i.e., >1 or >2 standard deviations (SD) below the mean).
- Study included a measure of association and statistical significance;
- Majority of study participants were healthy children without any existing developmental disabilities, neonatal morbidities, pathologies associated with cognitive deficits, or rare disorders.

All 258 studies included in the Ruiz et al. [48] review were examined for this analysis. Pearson’s r data were extracted from the original publications and converted to odds ratios (OR) for meta-analysis to determine the relationship between each factor and general cognitive ability (Table 1) [61]. Studies were excluded from this analysis if there was not enough statistical information to extract Pearson’s r, if there were not enough of the same type of stressors for a statistical comparison, or if there were too many covariates included in the original analysis that could not be separated into distinct categories, or if there were no other ‘like’ stressors to group the covariates with. Overall, the lack of statistical information was the most common reason for exclusion. A total of 185 studies were used in this analysis.

#### 2.1.2. Data Grouping

The extracted data were organized into five categories that we refer to as “factors”. The five factor categories are: activities/behaviors, social, inherent, maternal, and chemical stressors. By including the maternal factors category, we were able to separate prenatal stressors from childhood stressors for specific analyses, as well as examine the relationship between maternal stressors and childhood cognitive ability. Since many of the stressors could be categorized into more than one factor group, we also provide the secondary factor categories. The factor groups and associated subgroupings are provided in Table 1.

### 2.2. Statistical Analyses

Analyses were conducted and figures created using R Studio (version 1.2.5033) (RStudio, Boston, MA, USA) with the tidyverse, ggplot2, and metafor packages [62,63,64,65]. The code used in this work is provided in the accompanying R-markdown document created with the rmarkdown package [66,67].

#### 2.2.1. Meta-Analysis

The factor groups and associated subgroupings were analyzed using the random-effects meta-analysis (RMA) with the restricted maximum likelihood (REML) estimator. The random-effects method was selected because the included studies are diverse and report different metrics of childhood cognitive ability with data acquired through both survey and measurement. Therefore, this method provides a more appropriate estimate of variance than other estimators [68,69].

The output of the meta-analysis is reported as an odds ratio (OR) since the OR is a measure of association between exposure to a stressor and an outcome and can be used to compare the odds of various exposures eliciting the outcome of interest [70]. When the OR = 1, this indicates that the exposure does not affect the odds of the outcome occurring. When the OR > 1, higher odds of the outcome occurring are indicated, and lower odds of the outcome are indicated when the OR < 1 [70]. When the range of values used to determine the OR crosses below 0 or above 1 it indicates the strength of the data in terms of ‘effect/no effect’ and shows the range of the data included in the OR determination. Due to the diversity of the data, we report the OR as an indication of the likelihood of an effect on childhood cognitive ability rather than an indication of positive/negative association, so as not to overstate the results of our analysis.

#### 2.2.2. Data Visualization—Violin Plots

To present the analyzed data, we used violin plots because these types of plots provide the opportunity to visualize the density of the ORs within our large dataset. In the violin plots, the factor group/subgroup is provided along the y-axis and the OR scale is provided along the x-axis. The violin width represents the distribution of the ORs in each analysis and the violin height represents the OR density. The widest section of each violin indicates where the probability is greatest of the OR being that value based on the included data. The dots in the center of the violin represent the mean OR and the error bars represent the mean standard error (SE) from that dataset. The included reference count, lines of extracted data (i.e., sampling events), and collective sample size (i.e., individuals) from the included references/sampling events are included in Table 1 for reference.

#### 2.2.3. Sensitivity Analysis

Data robustness was examined post-hoc using seven meta-analytic estimation methods [71]. The seven estimation methods were the REML, the maximum likelihood (ML), the fixed-effects (FE), the Hedges (HE), the DerSimonian–Laird (DL), the empirical Bayesian (EB), and the Paul–Mandel (PM) estimators. Data robustness is expressed as a percentage that reflects the number of estimators that yielded ORs within 1% of each other, which is below the 10% threshold generally used (Table 2).

#### 2.2.4. Publication Bias

The risk of publication bias in the meta-analyses was estimated using two methods for comparison to the log OR from each original study: the inverse number of participants (1/*n*) and the standard error (SE). Since there is a correlation between the SE and the magnitude of the OR (which can lead to inflated type 1 error), we used the 1/*n* method of bias assessment. However, the results of both methods are presented, as the SE method is more common in the literature [72,73,74] (Table 2). We present these two statistical methods rather than the funnel plot that is often used with meta-analyses as the data included herein are quite diverse, and may lead to an incorrect assumption about the data based on the 1/SE calculation, which can also leads to an inflated type 1 error rate [75,76].

## 3. Results and Discussion

A total of 185 references from the Ruiz et al. [48] scoping review were included in this meta-analysis. From these 185 references, we extracted 408 maternal stressors, 245 inherent characteristics, 128 activities and behaviors, 125 social stressors, and 110 chemical stressors (Table 1). Stressors were grouped into either factor or stressor level groups for statistical analyses. Within each factor, there were at least three exposure routes composed of more specific stressors, which also included at least three sampling events. All stressor groups were statistically assessed using the RMA method, except for nitrogen dioxide (NO_2_) since two of the three sampling events were extracted from the same publication.

Stressors were grouped according to life stage (i.e., prenatal, childhood) and the results are presented longitudinally for ease of comprehension. Prenatal exposures include maternal factors and factors inherent to the child, since we believe that child-specific inherent characteristics are linked to maternal stressors experienced during pregnancy. Childhood factors include activities and behaviors and social and chemical stressors experienced after birth and span the birth–toddler life stages [17].

Analyses to determine publication bias were conducted on each factor group individually and no statistically significant publication bias was detected (Table 2). Lack of publication bias is most likely due to the inclusion of data with both positive and negative relationships to childhood cognitive ability rather than only positive-skewed data. The robustness of the included data is a testament to the number of publications included in the original scoping review, such that inclusion of a wide range of stressors resulted in a robust analysis regardless of the estimator used. Sensitivity analysis revealed robust sensitivity of the data in the meta-analyses. These metrics attest to the quality of the included data, with large sample sizes and included numerous sampling events to provide robust, unbiased results (Table 2).

### 3.1. Prenatal Exposures

The prenatal data presented here includes maternal factors that span the preconception and prenatal life stages and child-specific inherent characteristics that are determined during the prenatal period [17].

#### 3.1.1. Maternal Factors

There were 84 references with maternal factors, including 408 sampling events of several types of stressors: substance use (147 sampling events), socioeconomic metrics (139 sampling events), and maternal health (122 sampling events) [29,77,78,79,80,81,82,83,84,85,86,87,88,89,90,91,92,93,94,95,96,97,98,99,100,101,102,103,104,105,106,107,108,109,110,111,112,113,114,115,116,117,118,119,120,121,122,123,124,125,126,127,128,129,130,131,132,133,134,135,136,137,138,139,140,141,142,143,144,145,146,147,148,149,150,151,152,153,154,155,156,157,158,159,160]. Each group included several individual stressors. Substance use included alcohol (amount; 60 sampling events), cigarettes (38 sampling events), alcohol (binging; 19 sampling events), cocaine (18 sampling events), and other narcotics (12 sampling events). Socioeconomics included education (71 sampling events), income (29 sampling events), employment (15 sampling events), home location (13 sampling events), language (seven sampling events), resources (six sampling events), technology access (six sampling events), and home ownership (five sampling events). Maternal health included age (31 sampling events), mental health (24 sampling events), body mass index (BMI, 22 sampling events), thyroid health (20 sampling events), stress (20 sampling events), and cortisol (10 sampling events). When all maternal stressors were examined in the factor-level RMA, the OR was 1.04 (*p* ≤ 0.001; Table 1) which indicates that the factors are associated with a 4% greater likelihood of effect on childhood cognitive outcomes compared to when the factors are absent. The individual substance abuse stressors are discussed in the supplemental text.

##### Socioeconomics

**Figure 1:**
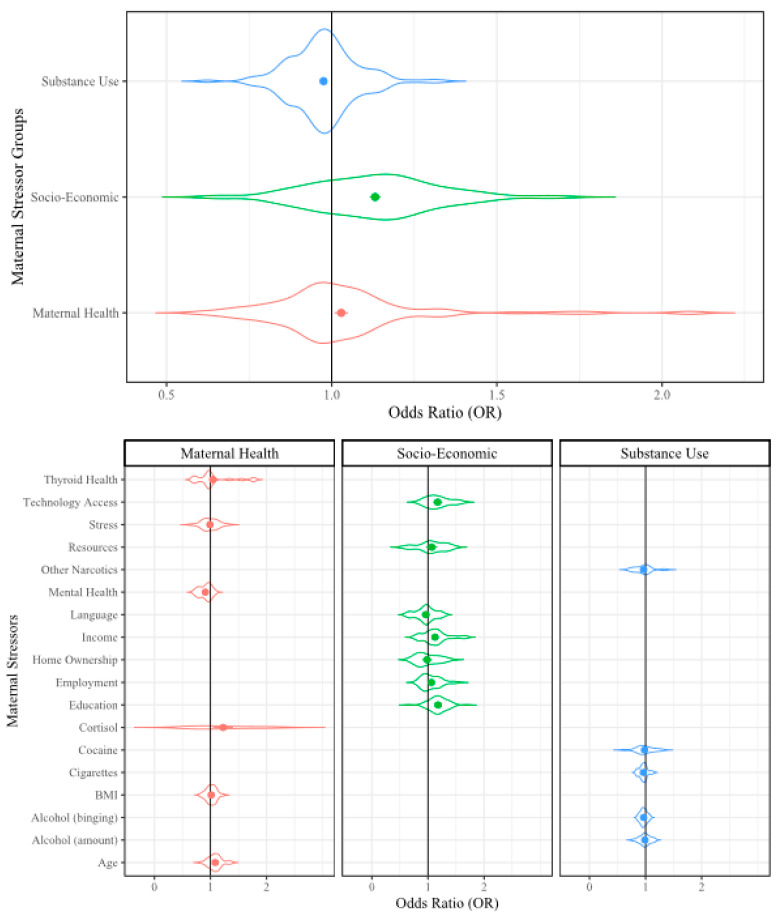
Violin plot of the maternal stressors that may influence cognitive development. Violin width represents the distribution of the Odds Ratios (OR), the height represents density of the ORs; dots represent mean OR, error bars represent mean standard error (SE). BMI denotes body mass index.

When stressors within the maternal factor group were analyzed separately, the socioeconomic stressors were observed to have the greatest impact on childhood cognitive ability, with a 13% increase in likelihood of effect indicated (OR = 1.13, *p* ≤ 0.001; Table 1). The stressors included in the socioeconomic group had varying amounts of influence on childhood cognitive ability, but all were statistically significant (Figure 1). Maternal education (OR = 1.18, *p* ≤ 0.001), technology access (OR = 1.17, *p* ≤ 0.01), and income (OR = 1.13, *p* ≤ 0.001) were observed to have the greatest likelihood of effect on childhood cognition, ranging from 13–18% increased likelihood. Employment (OR = 1.06, *p* ≤ 0.001) and resources (OR = 1.06, *p* ≤ 0.01) were observed to have a 6% increased effect on childhood cognition. On the other hand, home ownership (OR = 0.99, *p* ≤ 0.05) and language spoken (OR = 0.96, *p* ≤ 0.001) were observed to have a 1% and 9% decreased likelihood of effect on childhood cognitive ability (Table 1).

The greater impact observed for education, technology access, income, employment, and resources were logical as these stressors are interrelated. Interestingly, home ownership was observed to have a decreased impact even though it is also generally interrelated with the preceding stressors. The decreased impact of language on childhood cognitive ability is the most interesting, as bilingualism suggests proficient cognitive ability [161]. However, a child coming to an English-only setting from a non-English speaking household may struggle to adapt and learn skills taught in English as well as lose proficiency in their native language [161]. Further investigation is needed to determine whether or not a new language is beneficial or detrimental to early childhood cognition [162].

##### Maternal Health

The maternal health stressor group collective analysis revealed that these stressors have a 4% increased likelihood to influence childhood cognitive ability (OR = 1.04, *p* ≤ 0.001; Table 1, Figure 1). However, when the stressors were analyzed separately, greater detail was revealed. Maternal age (OR = 1.08, *p* ≤ 0.001), thyroid health (OR = 1.05, *p* ≤ 0.001), BMI (OR = 1.01, *p* ≤ 0.001), and cortisol concentration related to physiological stress (OR = 1.08, *p* ≤ 0.01) influenced childhood cognitive ability with increases between 1% and 8%, while stressors including mental health (OR= 0.91, *p* ≤ 0.001), and perceived stress (OR = 0.97, *p* ≤ 0.001) were statistically significant but had a decreased likelihood of influence compared to no exposure (Table 1).

It is well recognized that maternal age is directly related to adverse neonatal outcomes, so it is not surprising that maternal age influenced childhood cognitive ability [163,164]. Recent evidence suggests that thyroid disease is also associated with adverse pregnancy outcomes, and we observed that general thyroid health had an increased impact on childhood cognitive ability [165,166]. Similarly, both low and high maternal BMI are known to affect pregnancy outcomes, and the statistically significant relationship between maternal BMI and childhood cognition observed here could be further explored to determine underlying mechanisms [167,168]. Maternal mental health has been shown to be related to the child’s mental health outcomes, but it appears to be less of an influence for childhood cognitive ability [169].

Interestingly, both cortisol-measured and perceived stress were statistically significant but had opposite likelihoods of impact on childhood cognitive ability compared to no exposure to stress (*p* ≤ 0.001 and *p* ≤ 0.01, respectively). There is not always a correlation between elevated cortisol and perceived stress. In this study, cortisol measurements were observed to have a greater impact on childhood cognitive ability than perceived stress (Table 1). Since cortisol level is a physiological indicator of stress it is logical that this variable would be more closely related to childhood cognition. However, the significant relationship observed for perceived stress suggests that cognitive development is not solely influenced by physiology, but also by the emotional and mental environment associated with child development [11,21,60].

#### 3.1.2. Inherent Characteristics

There were 65 references that examined child-specific inherent characteristics. These references included: anthropometry (138 sampling events), birth outcomes (67 sampling events), and child health (40 sampling events) [29,36,79,81,82,83,84,88,89,97,99,100,101,102,103,104,106,107,108,113,115,116,119,120,122,123,126,127,128,133,136,137,138,139,142,143,146,147,148,149,150,152,153,155,156,157,159,170,171,172,173,174,175,176,177,178,179,180,181,182,183,184,185,186,187,188,189,190,191,192,193,194,195,196,197,198,199,200,201,202,203,204,205,206,207,208,209,210,211,212,213,214,215,216,217]. Stressors included in the anthropometry group were sex (63 sampling events), birth weight (47 sampling events), body length at birth (12 sampling events), head size at birth (10 sampling events), and fetal growth (6 sampling events). Birth outcomes included parity (24 sampling events), preterm birth (21 sampling events), gestational age (16 sampling events), delivery method (three sampling events), and if multiple births occurred (three sampling events). Child health included medical history (18 sampling events), genetic disorders (15 sampling events), and iron deficiency (seven sampling events). When all child-specific inherent characteristics were examined together in the factor-level RMA, the OR was 1.03 (*p* ≤ 0.001; Table 1) which indicated to us that the included characteristics were associated with a 3% greater effect on childhood cognitive outcomes than when the characteristics were absent. The individual child health stressors are discussed in the supplemental text.

##### Anthropometry and Birth Outcomes

**Figure 2:**
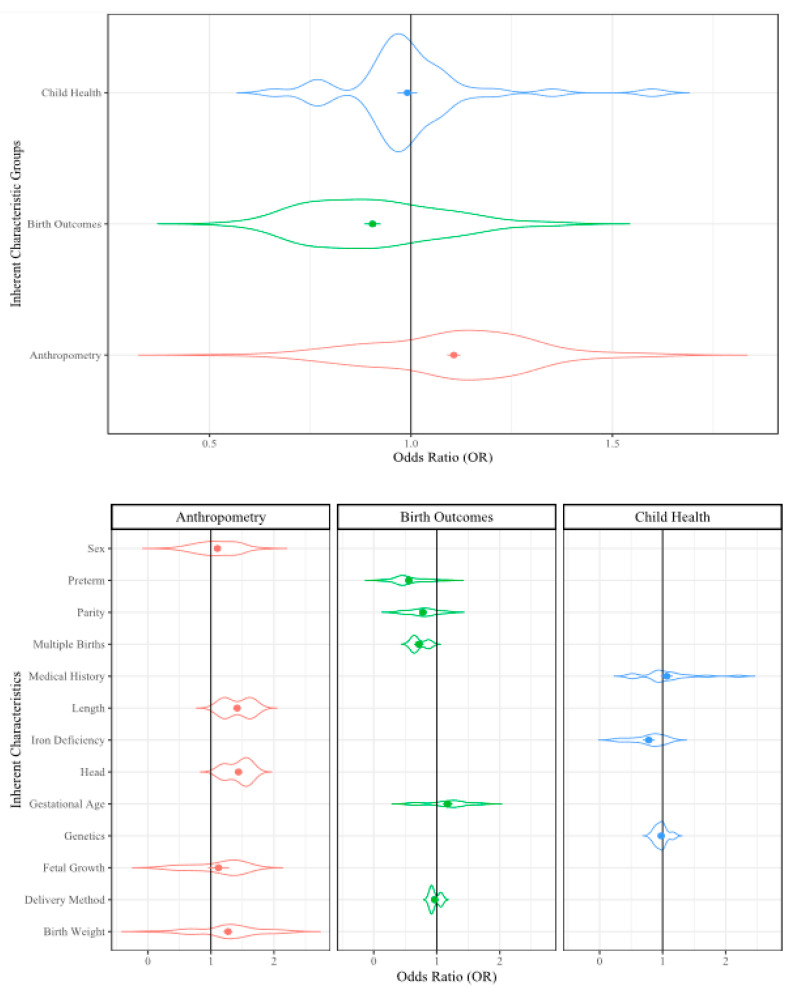
Violin plot of the inherent characteristics that may influence cognitive development. Violin width represents the distribution of the Odds Ratios (OR), the height represents density of the ORs; dots represent mean OR, error bars represent mean SE.

All anthropometrics were observed to be associated with a 10% increase in effect on childhood cognitive ability when combined in the RMA (OR = 1.1, *p* ≤ 0.001; Table 1, Figure 2). When individual stressors were considered, all were statistically significant (Table 1). Head circumference at birth was observed to have the greatest likelihood of impact on childhood cognitive ability with a 22% increase (OR = 1.22, *p* ≤ 0.001). Body length at birth (OR = 1.21, *p* ≤ 0.001) and birth weight (OR = 1.14, *p* ≤ 0.001) were observed to have 21% and 14% increases in likelihood of effect, respectively. Fetal growth was also observed to be influential to cognitive ability with a 6% increase in effect (OR = 1.06, *p* ≤ 0.01). These four stressors are interrelated, as well as related to the birth outcomes stressors: gestational age (OR = 1.11, *p* ≤ 0.001), preterm birth (OR = 0.78, *p* ≤ 0.001), and parity (OR = 0.89, *p* ≤ 0.001). These stressors are all related to the length of a pregnancy, the mother’s diet during pregnancy, previous pregnancies, and a number of other maternal stressors including age and BMI [9,218]. Low birth weight and preterm birth are related and known to result from risky pregnancies, so it is logical that they are all statistically significant in relation to decreased cognitive ability [219]. The most surprising result we observed within these groups was the high statistical significance that sex had in the RMA analysis (OR = 1.05, *p* ≤ 0.001; Table 1). Further investigation revealed that cognitive development is linked to sex hormone production and maturation rate. Since the original studies examined males and females of the same age, the additional dimension of this stressor may not have been captured in those studies and may have led to the drastic difference between the sexes in this analysis [220,221,222].

When all birth outcome stressors were examined together, they were observed to decrease the likelihood of influencing childhood cognitive development (OR = 0.91, *p* ≤ 0.001; Table 1, Figure 2). Gestational age was the only stressor that was observed to increase the likelihood of influencing childhood cognitive development (OR = 1.11, *p* ≤ 0.001). Both preterm birth (OR = 0.78 *p* ≤ 0.001), and parity (OR = 0.89, *p* ≤ 0.001) were observed to decrease the likelihood of influencing cognition. The decreasing influence of preterm birth was unexpected, particularly since related birth characteristics were observed to be increasingly influential. This observation may be due to the gestational timing of neural connections in the developing brain. Beginning at 20 weeks into pregnancy the neural circuitry begins to form and by the end of 35 weeks the most pertinent pieces of the neural network are in place. The transition through week 40 may not be relevant to whether the child is still in gestation or has been born, as many of these connections are related to sensory driven activity [223]. Delivery method (OR = 0.98) and multiple births (OR = 0.86) were not observed to be statistically significant and these stressors may be of less concern to cognitive development.

### 3.2. Childhood Exposures

Stressors included in the childhood exposure analysis are those likely to occur and be measured/reported during the childhood life stages (i.e., birth, infancy, toddler), specifically activities and behaviors and social and chemical stressors [17]. The childhood stressor results are presented longitudinally and begin where the prenatal stressors end. First, chemical stressors that children can be exposed to throughout all life stages are examined. Then, activities and behaviors that begin during birth and infancy stages are examined. Lastly, social stressors that arise during the toddler stage, including childcare stressors, are presented.

#### 3.2.1. Chemical Stressors

**Figure 3:**
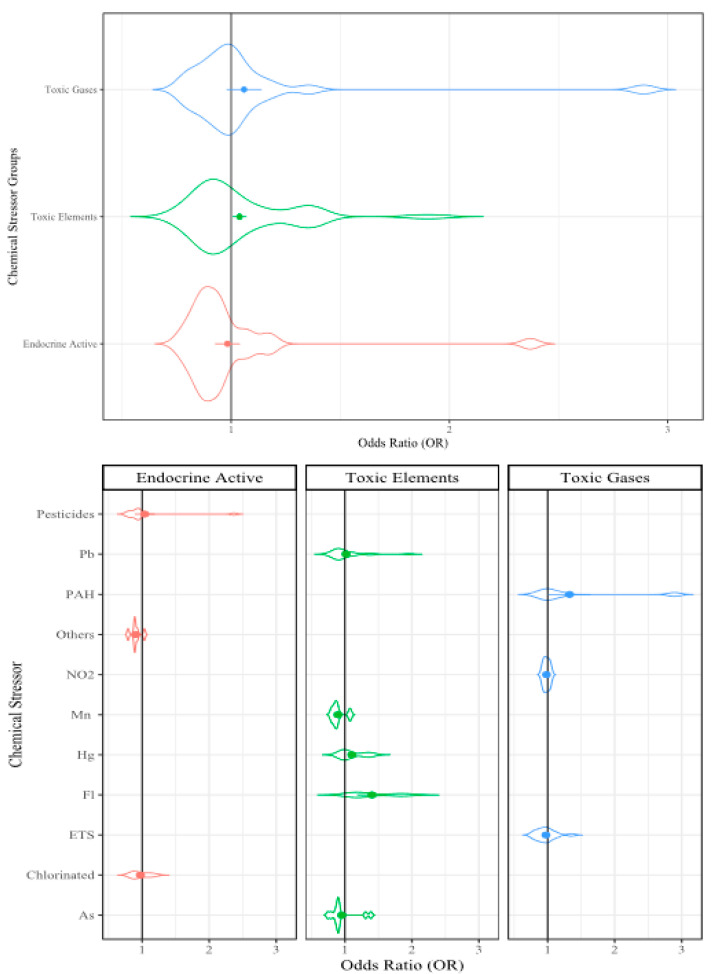
Violin plot of the chemical stressors that may influence cognitive development. Violin width represents the distribution of the Odds Ratios (OR), the height represents density of the ORs; dots represent mean OR, error bars represent mean SE.

The chemical stressors examined in this analysis included 41 references with 110 sampling events [45,95,100,103,106,113,116,120,121,123,132,133,136,138,139,148,149,150,155,156,157,186,200,207,211,212,213,224,225,226,227,228,229,230,231,232,233,234,235,236,237,238]. The chemical stressor group included toxic elements (58 sampling events), endocrine-active compounds (27 sampling events), and toxic gases (25 sampling events). The toxic element stressors included lead ((Pb), 23 sampling events), mercury ((Hg), 16 sampling events), arsenic ((As), 11 sampling events), manganese ((Mn), five sampling events), and fluoride ((Fl), three sampling events). The toxic gases stressors were environmental cigarette/tobacco smoke ((ETS), 16 sampling events), polycyclic aromatic hydrocarbons ((PAHs), six sampling events), and nitrogen dioxide ((NO_2_), three sampling events). The endocrine-active compounds included chlorinated chemicals ((polychlorinated biphenyls, PCBs; hexachlorobenzene, HCB; trichloropyridinol, TCPy); 11 sampling events), pesticides ((pyrethroids, Mirex, organophosphates); 10 sampling events), and others ((polybrominated diphenyl ethers, PBDEs; phthalates); six sampling events). Collectively, the chemical stressors were observed to have a 5% increase of influence on childhood cognitive ability (OR = 1.05, *p* ≤ 0.001; Table 1, Figure 3).

##### Toxic Elements

The toxic element stressors were observed to have a 4% increase in the likelihood of impacting childhood cognitive ability when examined together (OR = 1.04, *p* ≤ 0.001; Table 1). Heavy metal exposures (Hg, Pb, As, and cadmium (Cd)) are known risk factors for cognitive dysfunction as they interfere with neurotransmitter receptors in the brain [239]. When examined individually, Hg resulted in a 10% increase in impacting childhood cognition (OR = 1.10, *p* ≤ 0.001), which is not unexpected as Hg is a known neurotoxicant. Lead was also observed to have a slight (3%) increase in impact to cognition (OR = 1.03, *p* ≤ 0.001), and has been widely described to cause cognitive delays [240]. Fluoride was observed to have the greatest increase in impacting cognitive ability (OR = 1.40, *p* ≤ 0.05) and it is often reported to affect memory and cause cognitive deficits [241]. Both Mn (OR = 0.90, *p* ≤ 0.05), and As (OR = 0.95, *p* ≤ 0.01) were observed to decrease the likelihood of influencing childhood cognition (10% and 5%, respectively). While both Mn and As have been associated with cognitive impairment, the studies we examined in this analysis reported the Mn and As concentrations from water samples, while the other toxic elements were reported in biological samples [156,210,234,238,242]. These results highlight the discrepancies that can occur between different sampling methods and support the idea that the most direct method of sampling, in this case biological fluid samples as opposed to drinking water samples, will provide the most accurate measures for comparison.

##### Toxic Gases

When examined collectively in the RMA, the toxic gases were observed to have a 6% increase in likelihood of impacting childhood cognitive abilities (OR = 1.06, *p* ≤ 0.001; Table 1). When PAHs were examined, they had a 35% increase in their likelihood to influence childhood cognitive ability compared with when the toxic gases were not encountered (OR = 1.35, *p* ≤ 0.001). ETS was observed to have a 3% decrease in the likelihood of affecting childhood cognitive ability (OR = 0.97, *p* ≤ 0.001). The relationship between exposure to PAHs and neurodevelopment, cognitive development, and learning abilities has been described in a variety of epidemiological studies [8,243,244]. As PAHs comprise part of the chemical mixture of ETS, similar inverse relationships between exposure and cognition have been reported [245]. As both PAHs and ETS are widespread, more epidemiological data needs to be collected to elucidate causative relationships.

##### Endocrine-Active Stressors

The endocrine-active stressors resulted in a 1% decrease in their impact on childhood cognitive ability when analyzed together in the RMA (OR = 0.99, *p* ≤ 0.001; Table 1). When analyzed separately, only the pesticides had a significant increase in their effect on childhood cognition (OR = 1.05, *p* ≤ 0.001). Both the chlorinated compounds (OR = 0.97 *p* ≤ 0.01) and the PBDE/phthalate (OR = 0.92, *p* ≤ 0.05) groups resulted in decreases in the likelihood of an impact. The relationship between the endocrine system and cognitive ability has been well documented including the various modes of action through the endocrine system [246,247]. Interestingly, recent studies have reported that prenatal rather than childhood exposure to endocrine active compounds has a greater effect on childhood behavior [248,249,250]. The differences observed between the classes of endocrine-active chemicals in this work, as well as the differences reported between prenatal and childhood exposures, highlight the need for further examination of the specific relationships and modes of action that are relevant to childhood cognitive ability.

#### 3.2.2. Activities and Behaviors

**Figure 4:**
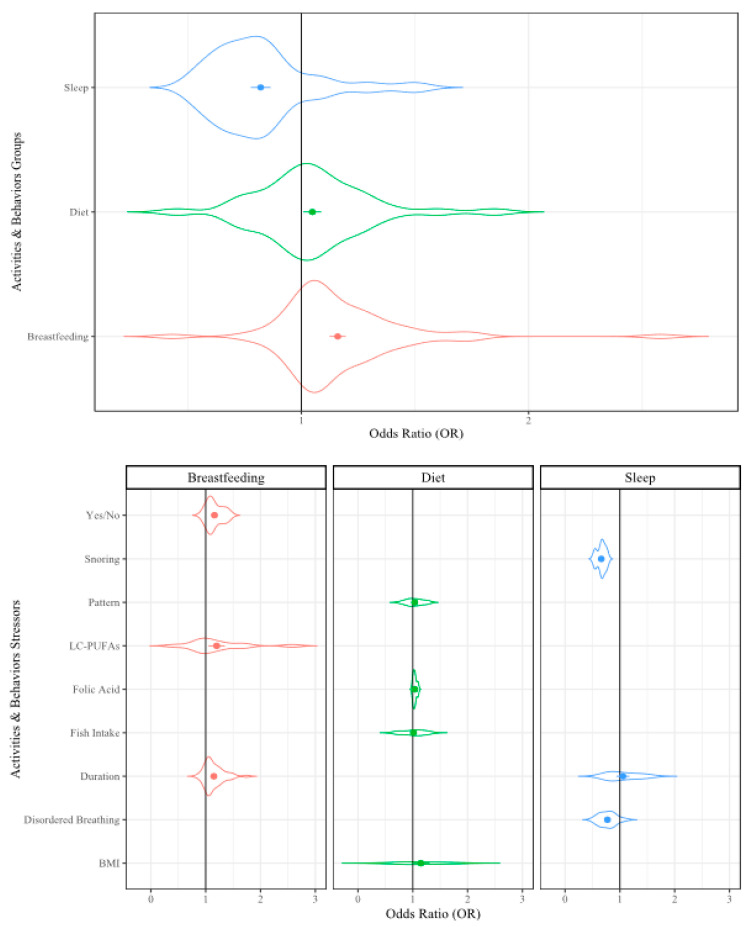
Violin plot of the activities/behaviors that may influence cognitive development. Violin width represents the distribution of the Odds Ratios (OR), the height represents density of the ORs; dots represent mean OR, error bars represent mean SE.

There were 41 references containing 128 sampling events included in the analysis of activities and behaviors [13,30,36,79,97,98,99,120,126,128,136,140,147,148,149,150,155,159,171,172,183,211,251,252,253,254,255,256,257,258,259,260,261,262,263,264,265,266,267,268,269,270]. The groups were breastfeeding behavior (63 sampling events), diet patterns (39 sampling events), and sleep characteristics (26 sampling events). The stressors included in breastfeeding behavior were duration of breastfeeding (44 sampling events), the presence of the long chain polyunsaturated fatty acids (LC-PUFAs) commonly associated with breastfeeding (13 sampling events), and if breastfeeding was conducted at all (yes/no; six sampling events). The dietary group included amount of fish consumed (16 sampling events), dietary pattern (10 sampling events), childhood BMI (eight sampling events), and folic acid supplementation (five sampling events). The sleep characteristics included were sleep disordered breathing (16 sampling events), sleep duration (six sampling events), and snoring behavior (four sampling events). When all activity and behavior stressors were analyzed collectively at the factor-level, we observed a 5% increase in the likelihood of effect on childhood cognitive ability (Table 1, Figure 4). The individual sleep stressors are discussed in the supplemental text.

##### Breastfeeding

When all breastfeeding stressors were examined together, there was a 16% increase in likelihood of effect on childhood cognitive ability (OR = 1.16, *p* ≤ 0.001; Table 1, Figure 4). When the stressors were examined individually, the LC-PUFAs were observed to have the greatest effect with a 20% increase (OR = 1.20, *p* ≤ 0.001; Table 1). If breastfeeding occurred at all was the next most impactful stressor with a 16% increase in effect on cognitive ability (OR = 1.16, *p* ≤ 0.001). Duration of breastfeeding was observed to have a 15% increase in effect on childhood cognitive ability (OR= 1.15, *p* ≤ 0.001). The relationship between these three stressors and childhood cognitive ability is in agreement with what has been reported in the literature as breastfeeding provides vitamins and nutrients necessary for healthy development [271,272]. Interestingly, when LC-PUFAs were supplemented into a formula-based diet, no improvement in general cognitive ability in infants was reported, but when specific cognitive behaviors were assessed, the supplemented children had improved visual attention and problem solving skills [273]. Children that received supplemented formula were also faster at decision-making by age 6 years than those that received non-supplemented formula [274,275]. Our results support the positive relationship between LC-PUFAs and general cognitive ability in childhood that has been previously documented in the peer-reviewed literature.

##### Diet

Dietary stressors included BMI, fish intake, folic acid, and diet pattern. When all dietary stressors were analyzed in the RMA, a 5% increase in the likelihood to effect childhood cognitive ability was observed (OR = 1.05, *p* ≤ 0.001; Table 1, Figure 4). When each stressor was examined separately, childhood BMI had a 14% increase in influencing cognitive ability (OR = 1.14, *p* ≤ 0.001). Dietary pattern (OR = 1.03, *p* ≤ 0.001), folic acid supplementation (OR = 1.04, *p* ≤ 0.05), and fish intake (OR = 1.01, *p* ≤ 0.01) all had significant increases in their likelihood to influence childhood cognitive ability. These stressors are components of a healthy diet, which influences BMI. Having a healthy diet that includes the proper amounts of vitamins and minerals has been shown to be important for childhood cognitive development [276]. The relationship between BMI and cognition is likely related to the dietary nutrients that are part of a healthy diet and missing from diets that include many processed foods, sodium, and fats [276]. However, the relationship between diet and BMI in early childhood is not clear, so future research could focus on this area to better understand childhood cognitive ability [277,278].

#### 3.2.3. Social Factors

**Figure 5:**
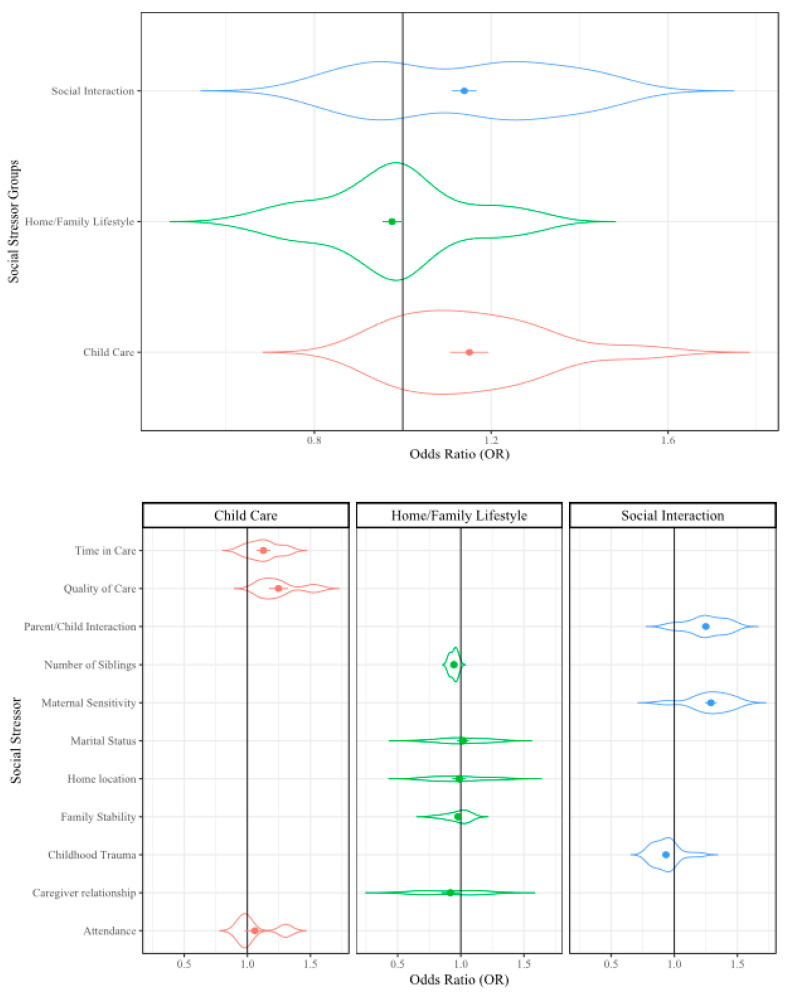
Violin plot of the social stressors that may influence cognitive development. Violin width represents the distribution of the Odds Ratios (OR), the height represents density of the ORs; dots represent mean OR, error bars represent mean SE.

The analysis of social stressors was composed of 33 references that included 125 sampling events related to childhood cognitive ability [29,80,83,94,98,101,120,122,126,127,132,136,137,139,142,150,152,153,155,158,173,176,181,189,210,211,256,278,279,280,281,282,283]. The individual stressors examined within the social stressors factor group were social interactions (57 sampling events), home/family lifestyle (57 sampling events), and childcare (14 sampling events; Table 1). The social interaction subgroup included the stressors: parent/child interaction (24 sampling events), childhood trauma (21 sampling events), and maternal sensitivity (11 sampling events). The home/family lifestyle subgroup included maternal marital status (14 sampling events), home location (e.g., rural, urban, metro; 13 sampling events), family stability (11 sampling events), relationship of the child to their caregiver (nine sampling events), and number of siblings a child has (eight sampling events). The childcare subgroup included quality of care (five sampling events), child’s time in care (five sampling events), and attendance at care (four sampling events). When all social stressors were examined together, a 7% increase in impact to childhood cognitive ability was observed (OR = 1.07, *p* ≤ 0.001; Table 1, Figure 5). The individual home/family lifestyle stressors are discussed in the supplemental text.

##### Social Interactions

When the social interaction stressors were analyzed together in the RMA, a 14% increase in the influence on childhood cognitive ability was observed (OR = 1.14, *p* ≤ 0.001; Table 1, Figure 5). Upon individual analysis, maternal sensitivity (OR = 1.29, *p* ≤ 0.001) and parent/child interaction (OR = 1.25, *p* ≤ 0.001) were observed to significantly increase the influence on childhood cognitive ability. Maternal sensitivity resulted in a 29% increase and parental interaction resulted in a 25% increase (Table 1). The large increase in the percent impact highlights the importance of each stressor in influencing childhood cognitive ability. Childhood traumatic experiences were observed to have a significant decrease in influencing cognition (OR = 0.93, *p* ≤ 0.001). The low likelihood of a relationship between childhood traumatic experiences and cognition was unexpected as it has been previously observed to be highly correlated to childhood mental health outcomes [169]. The notion that social interaction is related to childhood cognitive development has been well explored, but specific causative measures have been challenging to identify [27,284,285]. Our reported dichotomy between the impacts of maternal emotionality/parental interaction and childhood trauma on childhood cognitive ability may further elucidate the complex interactions between social stressors and cognition.

##### Childcare

When analyzed collectively in the RMA, childcare stressors were observed to have a 15% increase in their likelihood of influence on childhood cognitive ability (OR = 1.15, *p* ≤ 0.001; Table 1, Figure 5). Individual stressors included in the childcare group were also observed to have statistically significant increases in their likelihood of impact on childhood cognitive ability, but to varying percentages. The quality of childcare had the greatest likelihood of influence at 25% (OR = 1.25, *p* ≤ 0.001), time in childcare was second at 13% (OR = 1.13, *p* ≤ 0.01), while attendance was observed to be 7% (OR = 1.07, *p* ≤ 0.05). Several studies have shown that childcare quality is related to cognitive ability, with significant implications related to the quality of the environment (e.g., mentally stimulating, conducive for learning, comfortable), social interactions the child has with others, and the relationship the child has with the teacher or caregiver [27,286,287,288]. Childcare has been previously reported to be protective of cognitive ability in children that are also exposed to negative stressors (e.g., domestic violence, abuse) [289]. Relationships present in the literature also provide support for the percentages of influence observed in this study; the most significant stressor is the quality of care, followed by the time a child spends in the care environment, then followed by their general attendance at the childcare facility (Table 1, Figure 5).

##### Stressors Influencing Childhood Cognitive Ability

**Figure 6:**
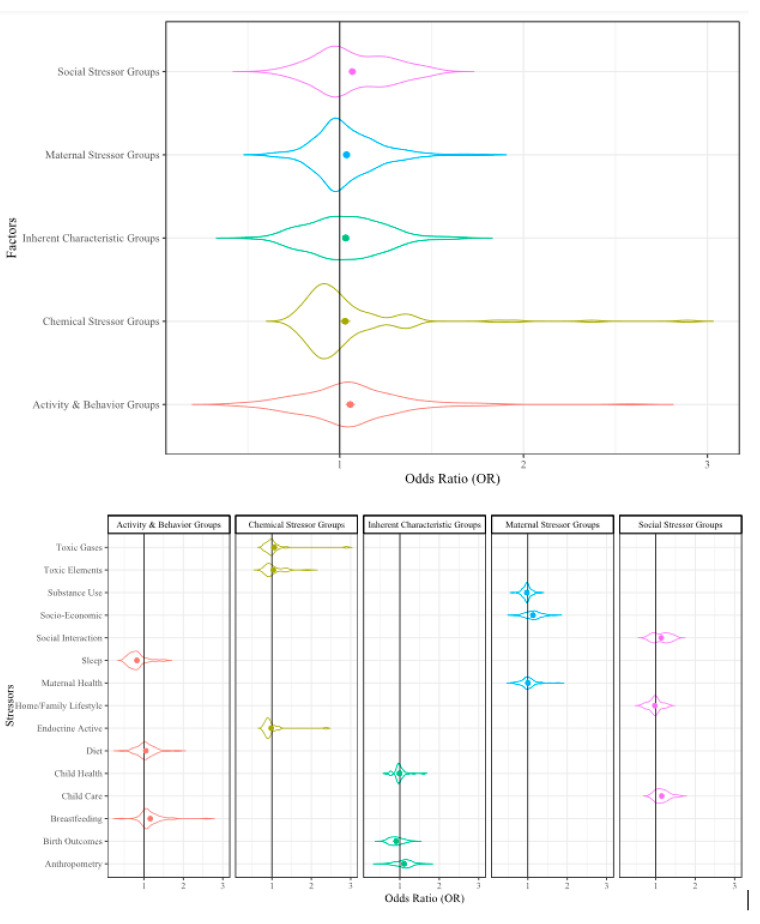
Violin plot of all factors that may influence cognitive development. Violin width represents the distribution of the Odds Ratios (OR), the height represents density of the ORs; dots represent mean OR, error bars represent mean SE.

Our analysis showed that many individual stressors had a small impact on childhood cognitive ability, but the collective impact of all chemical and non-chemical stressors experienced by children is not well understood, especially as it relates to cognitive ability. When all stressors were analyzed collectively, the result is a 4% increase in the likelihood of an influence on childhood cognition (OR = 1.04, *p* ≤ 0.001). When the five factor RMA results are compared, we observe a wide range of influence on childhood cognitive ability (Figure 6).

Our goal was to identify key stressors from the review conducted by Ruiz et al. [48]. To that end, this work identified 23 stressors that had a significant increase in their likelihood to influence childhood cognitive ability (by 10% or more) and 75 stressors that were statistically significant (Table 1).

The prenatal exposures observed to have the greatest impact of on childhood cognitive ability were maternal stressors related to the availability of information: education, income, and technology access. The inherent stressors with the greatest impact were those determined by the pregnancy environment and maternal behavior during pregnancy: head size, body length, and birth weight.

The childhood exposures observed to be most influential to cognitive ability were nutrition during infancy and throughout childhood, parental interaction, childcare, and toxic pollutant exposures (Table 1). Interestingly, each of these childhood stressors could also be attributed to maternal activities and behaviors (i.e., breastfeeding, childhood BMI, income), and are modifiable factors that can be changed by altered parental behavior. Our results highlight the importance of prenatal health, particularly how various stressors could impact a woman’s health during pregnancy, her activities and behaviors, and result in adversity for the child.

##### Limitations

A limitation of this work is that the meta-analysis included such diverse studies that specific “positive/negative” relationships could not be reported with certainty, only the “likelihood of impact”. While this provided the opportunity to include a wide array of primary research, additional research is required to elucidate the positive/negative effects of specific stressors on childhood cognitive ability.

The quality of a meta-analysis and the resulting conclusions are only as reliable as the included data. The results reported herein are the synthesis of high-quality observational and randomized control trial studies that met rigorous inclusion criteria. Specifically, that general cognitive outcome was measured using well established evidence-based assessments and expressed as a continuous variable or categorized as below average or significant cognitive delay (i.e., >1 or >2 standard deviations (SD) below the mean) [19]. Additionally, the majority of study participants were healthy children without any existing developmental disabilities, neonatal morbidities, or pathologies associated with cognitive deficits [48]. The most standardized and/or corrected data was extracted from the included references so that the data used in the meta-analysis would be as similar as possible. Variations in study context, goals, and inherent confounding factors were accounted for as much as possible using the random-effects meta-analysis model, and our language (i.e., likelihood of impact on childhood cognition) was chosen so as not to overstate the results of the analyses herein.

The challenge in pooling studies for meta-analyses lies in combining studies with differences in exposure assessment. Obtaining an agreed upon index of exposure to establish dose–response associations and a consensus in methodologies has proven difficult thus far. However, a consistent metric of exposure could lead to better understanding of the association between exposure and effect and increased certainty of effects on cognitive development. Establishing uniform metrics would enable the results of studies like those included here, and many others, to have a greater influence on risk management and policy making.

It should be noted that the studies included in this meta-analysis were primarily based in the United States (US), used data from primarily healthy children, and were from English speaking families. Due to these characteristics, extrapolating the results discussed herein to other areas of the world with chronic/ongoing stressors (i.e., chronic malnutrition, war, and other crises leading to the disruption of normal communities) may be challenging. The results discussing bilingualism and cognitive ability were also presented through an US-focused lens, where bilingualism is not as prevalent as other areas of the world. The global approach to multilingualism is quite different than the approach in the US; many countries have bilingualism as an inherent part of society, whereas the US does not, and this may have influenced our results. A greater understanding of the relationship between bilingualism and cognitive ability could be ascertained using global data.

## 4. Conclusions

Our goal was to determine if any key factors related to childhood cognitive ability could be identified from the stressors identified by Ruiz et al. [48]. Using the child health and well-being conceptual framework as a guide to life stage and stressor inter-relationships, this work identified key stressors in both the prenatal and early childhood life stages [17]. These stressors were maternal health and the mother’s ability to access information relevant to a healthy pregnancy (for the prenatal period), dietary nutrients (infancy), quality of social interaction (toddler), and exposure to toxic substances (throughout early childhood). In doing this analysis, we examined the relative impact of real-world exposures on cognitive development and have begun to understand the inter-relationships between exposures to chemical and non-chemical stressors and early childhood health outcomes.

Through this work we have elucidated the tremendous impact of maternal factors on childhood cognitive ability. The most significant maternal factors appear to be related to information accessibility and to maternal choices made during pregnancy. The child-specific inherent characteristics that we observed to be most impactful in influencing cognition are uncontrollable for the child but are at least partially controllable for the pregnant woman. Based on the data examined here, the more desirable behavioral, social, and chemical exposure factors may provide enhanced childhood cognitive abilities, and that quality may be related to maternal access to relevant information. As such, using available information regarding a healthy, full-term pregnancy, and making the recommended behavioral changes may improve cognitive outcomes related to exposures to prenatal stressors.

## Figures and Tables

**Table 1:** The odds ratio (OR) from the random-effects meta-analyses in this analysis are presented alongside the factor and subgroupings.

Prenatal Exposures						Childhood Exposures					
Primary Factor	Exposure + Variables	Studies	Sampling Events	Individuals	Secondary Factor	Primary Factor	Exposure + Variables	Studies	Sampling Events	Individuals	Secondary Factor
Maternal (1.04) ***	Socioeconomics (1.13) ***	55	139	113,976	Social	Activities & Behaviors (1.05) ***	Breastfeeding (1.16) ***	24	63	28,895	Maternal/Social
	Employment (1.06) ***	6	15	6818			Yes/No (1.16) **	6	6	3349	
	Education (1.18) ***	45	71	55,020			Duration (1.15) ***	16	47	23,789	
	Income (1.13) ***	19	29	28,235			LC-PUFAs (1.20) ***	3	13	1757	
	Language (0.96) *	5	7	2580			Diet (1.05) ***	17	39	46,179	
	Resources (1.06) **	5	6	3149			BMI (1.14) ***	3	8	1802	
	Home Ownership (0.99) *	3	5	17,418			Diet pattern (1.03) **	5	10	39,284	
	Technology Access (1.17) **	3	6	602			Folic Acid (1.04) *	4	5	1499	
	Maternal Health (1.02) ***	43	127	125,369	Inherent/Social		Fish Intake (1.01) ***	5	16	3594	
	Age (1.08) ***	25	31	25,100			Sleep (0.82) ***	11	26	3963	
	BMI (1.01) ***	10	22	52,271			Duration (1.06) **	3	3	657	
	Thyroid Health (1.05) ***	6	20	6121			Snoring (0.66)	4	4	356	
	Mental Health (0.91) ***	11	24	36,909			Disordered Breathing (0.77) **	8	16	2950	
	Stress (perceived) (0.97) ***	7	20	4917		Social (1.07) ***	Social Interaction (1.14) ***	18	56	43,081	Inherent/Maternal
	Stress (cortisol) (1.08) **	3	10	1496			Maternal Sensitivity (1.29) ***	7	11	2092	
	Substance Use (0.97) ***	39	147	159,067	Social		Parent/Child Interact (1.25) ***	7	24	26,804	
	Alcohol (amount) (0.99) ***	19	60	64,292			Childhood Trauma (0.93) ***	6	21	14,185	
	Alcohol (binging) (0.96) ***	4	19	36,817			Home/Family (0.98) ***	27	55	119,554	
	Cigarettes (0.96) ***	25	38	51,251			Number of Siblings (0.94) **	6	8	17,393	
	Cocaine (0.98) ***	9	18	4218			Caregiver Relationship (0.92) **	5	9	4579	
	Other Narcotics (0.96) **	5	12	2489			Family Stability (0.98) ***	7	11	41,287	
Inherent (1.03) ***	Anthropometry (1.1) ***	84	138	111,310	Maternal		Marital Status (1.01) ***	11	14	53,001	
	Head Size (1.22) ***	10	10	4111			Home Location (0.99) ***	4	13	3294	
	Body Length (1.21) ***	9	12	17,112			Childcare (1.15) ***	4	14	4144	
	Birth Weight (1.14) ***	37	47	43,129			Quality of Care (1.25) **	3	5	1349	
	Fetal Growth (1.06) **	3	6	2315			Time in Care (1.13) **	3	5	2251	
	Sex (1.05) ***	59	63	44,643			Attendance (1.07) *	4	4	4144	
	Birth Outcomes (0.91) ***	33	67	53,490		Chemical (1.05) ***	Toxic Gases (1.06) ***	16	25	10,640	Social/Activities & Behaviors
	Delivery Method (0.98)	3	3	2532			PAHs (1.35) ***	5	6	1514	
	Preterm (0.78) ***	11	21	16,558			Cigarette Smoke (0.97) ***	13	16	7017	
	Parity (0.89) ***	14	24	20,195			Toxic Elements (1.04) ***	25	58	25,754	
	Multiple Births (0.86)	3	3	8387			Fluorine (1.40) *	3	3	902	
	Gestational Age (1.11) ***	13	16	5818			Arsenic (0.95) **	5	11	12,249	
	Child Health (1.0) ***	18	40	63,969			Manganese (0.90) *	3	5	520	
	Medical History (1.03) ***	6	18	47,923			Lead (1.03) ***	11	23	6314	
	Iron Deficiency (0.89) *	6	7	1302			Mercury (1.10) ***	8	16	4769	
	Genetics (0.99) ***	4	15	14,744			Endocrine Active (0.99) ***	12	27	4621	
							Pesticides (1.05) ***	8	10	1498	
							Chlorinated (0.92) **	3	11	1835	
							PBDE/phthalates (0.92) *	3	6	1288	

*** *p* ≤ 0.001 ** *p* ≤ 0.01 * *p* ≤ 0.05; The number of studies indicates the number of references used in this analysis; The number of sampling events indicates the data points that were extracted from the included references; The number of individuals is the collective sample size for all included studies in each group/subgroup. PAH-polycyclic aromatic hydrocarbons; PBDE-polybrominated diphenyl ethers; BMI-body mass index; LC-PUFA-long chain polyunsaturated fatty acids

**Table 2:** The results of the sensitivity and publication bias analyses for the data analyzed in this publication. The Odds Ratio (OR) for each of the seven estimation methods are presented. The estimation methods were the restricted maximum likelihood (REML), the maximum likelihood (ML), the fixed-effects (FE), the Hedges (HE), the DerSimonian–Laird (DL), the empirical Bayesian (EB), and the Paul–Mandel (PM) estimators. Standard error is denoted by SE, and the inverse of the sample size is denoted by 1/n.

Factor	Sensitivity Analysis								Publication Bias	
	Meta-Analysis Estimation Model							Data Robustness	Method	*p*-Value
	REML (This Study)	FE	HE	DL	PM	EB	ML	(% ORs within 10%)		
Maternal	1.04	1.04	1.04	1.04	1.04	1.04	1.04	100%	SE	0.86
	OR Difference	0	0	0	0	0	0		1/n	0.88
	% Difference	0%	0%	0%	0%	0%	0%			
Inherent	1.03	1.03	1.03	1.03	1.03	1.03	1.03	100%	SE	0.76
	OR Difference	0	0	0	0	0	0		1/n	1.0
	% Difference	0%	0%	0%	0%	0%	0%			
Chemical	1.05	1.05	1.05	1.05	1.05	1.05	1.05	100%	SE	0.67
	OR Difference	0.0	0.0	0.0	0.0	0.0	0.0		1/n	0.72
	% Difference	0%	0%	0%	0%	0%	0%			
Behavioral	1.06	1.06	1.06	1.06	1.06	1.06	1.06	100%	SE	0.87
	OR Difference	0	0	0	0	0	0		1/n	0.89
	% Difference	0%	0%	0%	0%	0%	0%			
Social	1.10	1.10	1.10	1.10	1.10	1.10	1.10	100%	SE	0.63
	OR Difference	0	0	0	0	0	0		1/n	0.59
	% Difference	0%	0%	0%	0%	0%	0%			

## Supplementary Materials

The following are available online at https://www.mdpi.com/1660-4601/17/15/5451/s1.
