# Supplementary material for: A Meta-Analysis of Stressors from the Total Environment Associated with Children’s General Cognitive Ability
Source: Int J Environ Res Public Health. 2020 Jul 29;17(15):5451. doi: 10.3390/ijerph17155451 (PMC7432904; doi:10.3390/ijerph17155451)
Supplement: Supplementary file 1 [file ijerph-17-05451-s001.zip › Nilsen et al R Markdown.html]

Meta-Analysis, Bias Assessment, & Violin Plot Rmarkdown


# Meta-Analysis, Bias Assessment, & Violin Plot Rmarkdown

#### F. Nilsen; nilsen.frances@epa.gov Accompanying Rmarkdown file for *A Meta-Analysis of Stressors from the Total Environment Associated with Children’s General Cognitive Ability* IJERPH; vol; iss; pgs

---

# 1 Set R Environment

## 1.1 Open required packages.

Use *r::install.packages()* if the required packages are not previously installed.

```
library(openxlsx)
library(ggplot2)
library(tidyverse)
```

```
## -- Attaching packages ------------------------------------------------------------- tidyverse 1.2.1 --
```

```
## v tibble  2.1.3     v purrr   0.3.2
## v tidyr   0.8.3     v dplyr   0.8.3
## v readr   1.3.1     v stringr 1.4.0
## v tibble  2.1.3     v forcats 0.4.0
```

```
## -- Conflicts ---------------------------------------------------------------- tidyverse_conflicts() --
## x dplyr::filter() masks stats::filter()
## x dplyr::lag()    masks stats::lag()
```

```
library(metafor)
```

```
## Loading required package: Matrix
```

```
## 
## Attaching package: 'Matrix'
```

```
## The following object is masked from 'package:tidyr':
## 
##     expand
```

```
## Loading 'metafor' package (version 2.1-0). For an overview 
## and introduction to the package please type: help(metafor).
```

---

# 2 Load Data

## 2.1 Load data using the *openxlsx* package.

Use Data Appendix from the publication or use the file provided on EPA’s ScienceHub website. **[add links]**

---

# 3 Conduct Meta-Analyses

## 3.1 Run meta-analysis with the *metafor* package for each Factor.

### 3.1.1 All Cognitive Data Analysis:

```
rma(OR,V,data=AllCogDat, method="REML")
```

```
## Warning in rma(OR, V, data = AllCogDat, method = "REML"): Studies with NAs
## omitted from model fitting.
```

```
## 
## Random-Effects Model (k = 1015; tau^2 estimator: REML)
## 
## tau^2 (estimated amount of total heterogeneity): 0 (SE = 0.0417)
## tau (square root of estimated tau^2 value):      0
## I^2 (total heterogeneity / total variability):   0.00%
## H^2 (total variability / sampling variability):  1.00
## 
## Test for Heterogeneity:
## Q(df = 1014) = 51.7990, p-val = 1.0000
## 
## Model Results:
## 
## estimate      se     zval    pval   ci.lb   ci.ub 
##   1.0427  0.0304  34.2494  <.0001  0.9830  1.1024  *** 
## 
## ---
## Signif. codes:  0 '***' 0.001 '**' 0.01 '*' 0.05 '.' 0.1 ' ' 1
```

### 3.1.2 Individual Factor Code:

```
MaternRMA<- rma(OR, V, data=AllCogDat, subset=(Factors=="Maternal Stressor Groups"),method="REML")
InherentRMA<- rma(OR, V, data=AllCogDat,subset=(Factors=="Inherent Characteristic Groups"), method="REML")
BehavRMA<- rma(OR, V, data=AllCogDat, subset=(Factors=="Activity & Behavior Groups"), method="REML")
```

```
## Warning in rma(OR, V, data = AllCogDat, subset = (Factors == "Activity &
## Behavior Groups"), : Studies with NAs omitted from model fitting.
```

```
ChemRMA<- rma(OR, V, data=AllCogDat,subset=(Factors=="Chemical Stressor Groups"), method="REML")
SocialRMA<- rma(OR, V, data=AllCogDat,subset=(Factors=="Social Stressor Groups"), method="REML")
```

## 3.2 Run meta-analysis with the *metafor* package for each Exposure and Stressor/Variable within each Factor group.

### 3.2.1 Maternal Analysis Code:

```
SESRMA<- rma(OR, V, data=AllCogDat, subset=(Exposure=="Socio-Economic"), method="REML")
EmployRMA<- rma(OR, V, data=AllCogDat, subset=(Variable=="Employment"), method="REML")
EduRMA<- rma(OR, V, data=AllCogDat, subset=(Variable=="Education"), method="REML")
IncomeRMA<- rma(OR, V, data=AllCogDat, subset=(Variable=="Income"), method="REML")
LangRMA<- rma(OR, V, data=AllCogDat, subset=(Variable=="Language"), method="REML")
ResRMA<- rma(OR, V, data=AllCogDat, subset=(Variable=="Resources"), method="REML")
HomOwnRMA<- rma(OR, V, data=AllCogDat, subset=(Variable=="Home Ownership"), method="REML")
TechRMA<- rma(OR, V, data=AllCogDat, subset=(Variable=="Technology Access"), method="REML")

MatHealRMA<- rma(OR, V, data=AllCogDat, subset=(Exposure=="Maternal Health"), method="REML")
MAgeRMA<- rma(OR, V, data=AllCogDat, subset=(Variable=="Age"), method="REML")
MBMIRMA<- rma(OR, V, data=AllCogDat, subset=(Variable=="M-BMI"), method="REML")
AllThyRMA<- rma(OR, V, data=AllCogDat, subset=(Variable=="Thyroid Health"), method="REML")
MatMentalHealth<- rma(OR, V, data=AllCogDat, subset=(Variable=="Mental Health"), method="REML")
StressRMA<- rma(OR, V, data=AllCogDat, subset=(Variable=="Stress"), method="REML")
CortRMA<- rma(OR, V, data=AllCogDat, subset=(Variable=="Cortisol"), method="REML")

UseRMA<- rma(OR, V, data=AllCogDat, subset=(Exposure=="Substance Use"), method="REML")
AlcAmtRMA<- rma(OR, V, data=AllCogDat, subset=(Variable=="Alcohol (amount)"), method="REML")
AlcBinRMA<- rma(OR, V, data=AllCogDat, subset=(Variable=="Alcohol (binging)"), method="REML")
CigRMA<- rma(OR, V, data=AllCogDat, subset=(Variable=="Cigarettes"), method="REML")
CocaRMA<- rma(OR, V, data=AllCogDat, subset=(Variable=="Cocaine"), method="REML")
NarcotRMA<- rma(OR, V, data=AllCogDat, subset=(Variable=="Other Narcotics"), method="REML")
```

### 3.2.2 Inherent Analysis Code:

```
AnthroRMA<- rma(OR, V, data=AllCogDat, subset=(Exposure=="Anthropometry"), method="REML")
HeadRMA<- rma(OR, V, data=AllCogDat, subset=(Variable=="Head Size"), method="REML")
BodyRMA<- rma(OR, V, data=AllCogDat, subset=(Variable=="Body Length"), method="REML")
BirthWTRMA<- rma(OR, V, data=AllCogDat, subset=(Variable=="Birth Weight"), method="REML")
FetRMA<- rma(OR, V, data=AllCogDat, subset=(Variable=="Fetal Growth"), method="REML")
SexRMA<- rma(OR, V, data=AllCogDat, subset=(Variable=="Sex"), method="REML")

BirthOutRMA<- rma(OR, V, data=AllCogDat, subset=(Exposure=="Birth Outcomes"), method="REML")
DelivRMA<- rma(OR, V, data=AllCogDat, subset=(Variable=="Delivery Method"), method="REML")
TermRMA<- rma(OR, V, data=AllCogDat, subset=(Variable=="Preterm"), method="REML")
ParitRMA<- rma(OR, V, data=AllCogDat, subset=(Variable=="Parity"), method="REML")
MultlBirRMA<- rma(OR, V, data=AllCogDat, subset=(Variable=="Multiple Births"), method="REML")
GestAgeRMA<- rma(OR, V, data=AllCogDat, subset=(Variable=="Gestational Age"), method="REML")

ChHealRMA<- rma(OR, V, data=AllCogDat, subset=(Exposure=="Child Health"), method="REML")
MedHistRMA<- rma(OR, V, data=AllCogDat, subset=(Variable=="Medical History"), method="REML")
IronRMA<- rma(OR, V, data=AllCogDat, subset=(Variable=="Iron Deficiency"), method="REML")
GenetRMA<- rma(OR, V, data=AllCogDat, subset=(Variable=="Genetics"), method="REML")
```

### 3.2.3 Behavioral Analysis Code:

```
BfeedRMA<- rma(OR, V, data=AllCogDat, subset=(Exposure=="Breastfeeding"), method="REML")
```

```
## Warning in rma(OR, V, data = AllCogDat, subset = (Exposure ==
## "Breastfeeding"), : Studies with NAs omitted from model fitting.
```

```
BfeedYNRMA<-rma(OR, V, data=AllCogDat, subset=(Variable=="Yes/No"),method="REML")
```

```
## Warning in rma(OR, V, data = AllCogDat, subset = (Variable == "Yes/No"), :
## Studies with NAs omitted from model fitting.
```

```
BDuratRMA<-rma(OR, V, data=AllCogDat, subset=(Variable=="Duration"),method="REML")
PUFARMA<-rma(OR, V, data=AllCogDat, subset=(Variable=="LC-PUFAs"),method="REML")

DietRMA<- rma(OR, V, data=AllCogDat, subset=(Exposure=="Diet"), method="REML")
BMIRMA<-rma(OR, V, data=AllCogDat, subset=(Variable=="BMI"),method="REML")
PattRMA<-rma(OR, V, data=AllCogDat, subset=(Variable=="Pattern"),method="REML")
FolicRMA<-rma(OR, V, data=AllCogDat, subset=(Variable=="Folic Acid"),method="REML")
FishRMA<-rma(OR, V, data=AllCogDat, subset=(Variable=="Fish Intake"),method="REML")

SleepRMA<- rma(OR, V, data=AllCogDat, subset=(Exposure=="Sleep"), method="REML")
SDuratRMA<- rma(OR, V, data=AllCogDat, subset=(Variable=="S-Duration"), method="REML")
SnoringRMA<- rma(OR, V, data=AllCogDat, subset=(Variable=="Snoring"), method="REML")
SDBRMA<- rma(OR, V, data=AllCogDat, subset=(Variable=="Disordered Breathing"), method="REML")
```

### 3.2.4 Social Analysis Code:

```
SocIntRMA<- rma(OR, V, data=AllCogDat, subset=(Exposure=="Social Interaction"), method="REML")
MatSensRMA<- rma(OR, V, data=AllCogDat, subset=(Variable=="Maternal Sensitivity"), method="REML")
PatIntRMA<- rma(OR, V, data=AllCogDat, subset=(Variable=="Parent/Child Interaction"), method="REML")
TraumaRMA<- rma(OR, V, data=AllCogDat, subset=(Variable=="Childhood Trauma"), method="REML")

HomFamRMA<- rma(OR, V, data=AllCogDat, subset=(Exposure=="Home/Family Lifestyle"), method="REML")
SibsRMA<- rma(OR, V, data=AllCogDat, subset=(Variable=="Number of Siblings"), method="REML")
CaregivRMA<- rma(OR, V, data=AllCogDat, subset=(Variable=="Caregiver relationship"), method="REML")
MarStatRMA<- rma(OR, V, data=AllCogDat, subset=(Variable=="Marital Status"), method="REML")
FamStabilRMA<- rma(OR, V, data=AllCogDat, subset=(Variable=="Family Stability"), method="REML")
HomLocRMA<- rma(OR, V, data=AllCogDat, subset=(Variable=="Home location"), method="REML")

CareRMA<- rma(OR, V, data=AllCogDat, subset=(Exposure=="Child Care"), method="REML")
QualRMA<- rma(OR, V, data=AllCogDat, subset=(Variable=="Quality of Care"), method="REML")
TimeRMA<- rma(OR, V, data=AllCogDat, subset=(Variable=="Time in Care"), method="REML")
AttendRMA<- rma(OR, V, data=AllCogDat, subset=(Variable=="Attendance"), method="REML")
```

### 3.2.5 Chemical Analysis Code:

```
ToxGasRMA<- rma(OR, V, data=AllCogDat, subset=(Exposure=="Toxic Gases"), method="REML")
PAHRMA<- rma(OR, V, data=AllCogDat, subset=(Variable=="PAH"), method="REML")
ETSRMA<- rma(OR, V, data=AllCogDat, subset=(Variable=="ETS"), method="REML")

ToxEleRMA<- rma(OR, V, data=AllCogDat, subset=(Exposure=="Toxic Elements"), method="REML")
FLRMA<- rma(OR, V, data=AllCogDat, subset=(Variable=="Fl"), method="REML")
PbRMA<- rma(OR, V, data=AllCogDat, subset=(Variable=="Pb"), method="REML")
AsRMA<- rma(OR, V, data=AllCogDat, subset=(Variable=="As"), method="REML")
MnRMA<- rma(OR, V, data=AllCogDat, subset=(Variable=="Mn "), method="REML")
HgRMA<- rma(OR, V, data=AllCogDat, subset=(Variable=="Hg"), method="REML")


EndoRMA<- rma(OR, V, data=AllCogDat, subset=(Exposure=="Endocrine Active"), method="REML")
PestRMA<- rma(OR, V, data=AllCogDat, subset=(Variable=="Pesticides"), method="REML")
ChorRMA<- rma(OR, V, data=AllCogDat, subset=(Variable=="Chlorinated"), method="REML")
OthRMA<- rma(OR, V, data=AllCogDat, subset=(Variable=="Others"), method="REML")
```

---

# 4 Create Violin Plots

## 4.1 Create Violin Plots using *ggplot2* package.

### 4.1.1 All Cognitive Data Violin Plot

```
ggplot(AllCogDat, aes(Factors, OR, color=Factors))+
  geom_violin(trim=FALSE)+
  geom_hline(yintercept = 1)+
  labs(x="Factors", y="Odds Ratio (OR)")+
  stat_summary(fun.data=mean_se, geom="pointrange")+
  coord_flip()+
  theme_bw(14)+ theme(text=element_text(size=14, family="serif"))+theme(legend.position="none")
```

### 4.1.2 Maternal Data Violin Plot

```
ggplot(MaternalDat, aes(Maternal, OR, color=Maternal))+
  geom_violin(trim=FALSE)+
  geom_hline(yintercept = 1)+
  labs(x="Maternal Stressors Groups", y="Odds Ratio (OR)")+
  stat_summary(fun.data=mean_se, geom="pointrange")+
  coord_flip()+
  theme_bw(14)+ theme(text=element_text(size=14, family="serif"))+theme(legend.position="none")
```

### 4.1.3 Inherent Data Violin Plot

```
ggplot(InherentDat, aes(Inherent, OR, color=Inherent))+
  geom_violin(trim=FALSE)+
  geom_hline(yintercept = 1)+
  labs(x="Inherent Characteristic Groups", y="Odds Ratio (OR)")+
  stat_summary(fun.data=mean_se, geom="pointrange")+
  coord_flip()+
  theme_bw(14)+ theme(text=element_text(size=14, family="serif"))+theme(legend.position="none")
```

### 4.1.4 Behavioral Data Violin Plot

```
ggplot(BehavDat, aes(Behavior, OR, color=Behavior))+
  geom_violin(trim=FALSE)+
  geom_hline(yintercept = 1)+
  labs(x="Activity & Behavior Groups", y="Odds Ratio (OR)")+
  stat_summary(fun.data=mean_se, geom="pointrange")+
  coord_flip()+
  theme_bw(14)+ theme(text=element_text(size=14, family="serif"))+theme(legend.position="none")
```

### 4.1.5 Social Data Violin Plot

```
ggplot(SocialDat, aes(Social, OR, color=Social))+
  geom_violin(trim=FALSE)+
  geom_hline(yintercept = 1)+
  labs(x="Social Stressor Groups", y="Odds Ratio (OR)")+
  stat_summary(fun.data=mean_se, geom="pointrange")+
  coord_flip()+
  theme_bw(14)+ theme(text=element_text(size=14, family="serif"))+theme(legend.position="none")
```

### 4.1.6 Chemical Data Violin Plot

```
ggplot(ChemicalDat, aes(Chemical, OR, color=Chemical))+
  geom_violin(trim=FALSE)+
  geom_hline(yintercept = 1)+
  labs(x="Chemical Stressor Groups", y="Odds Ratio (OR)")+
  stat_summary(fun.data=mean_se, geom="pointrange")+
  coord_flip()+
  theme_bw(14)+ theme(text=element_text(size=14, family="serif"))+theme(legend.position="none")
```

---

# 5 Create Faceted Violin Plots

## 5.1 Create Faceted Violin Plots to show greater detail using *ggplot2* package.

### 5.1.1 All Cognitive Data Faceted Violin Plot

```
ggplot(AllCogDat, aes(Exposure, OR, color=Factors))+
  geom_violin(trim=FALSE)+
  geom_hline(yintercept = 1)+
  labs(x="Stressor", y="Odds Ratio (OR)")+
  stat_summary(fun.data=mean_se, geom="pointrange")+
  facet_grid(~Factors)+
    coord_flip()+
  theme_bw(14)+theme(text=element_text(size=14, family="serif"))+
  theme(legend.position="none", strip.text = element_text(size=14, colour="black"),
        strip.background = element_rect(fill="white", colour="black",size=1))
```

### 5.1.2 Maternal Data Faceted Violin Plot

```
ggplot(MaternalDat, aes(Variable, OR, color=Maternal))+
  geom_violin(trim=FALSE)+
  geom_hline(yintercept = 1)+
  labs(x="Maternal Stressors", y="Odds Ratio (OR)")+
  stat_summary(fun.data=mean_se, geom="pointrange")+
facet_grid(~Maternal)+
  scale_y_continuous(labels=scales::number_format(accuracy=1),breaks=0:2)+
  coord_flip()+
  theme_bw(14)+theme(text=element_text(size=14, family="serif"))+
  theme(legend.position="none", strip.text = element_text(size=14, colour="black"),
                      strip.background = element_rect(fill="white", colour="black",size=1))
```

### 5.1.3 Inherent Data Faceted Violin Plot

```
ggplot(InherentDat, aes(Variable, OR, color=Inherent))+
  geom_violin(trim=FALSE)+
  geom_hline(yintercept = 1)+
  labs(x="Inherent Stressors", y="Odds Ratio (OR)")+
  stat_summary(fun.data=mean_se, geom="pointrange")+
  facet_grid(~Inherent)+
  scale_y_continuous(labels=scales::number_format(accuracy=1,breaks=0:3))+
  coord_flip()+
  theme_bw(14)+theme(text=element_text(size=14, family="serif"))+
  theme(legend.position="none", strip.text = element_text( size=14, colour="black"),
        strip.background = element_rect(fill="white", colour="black",size=1))
```

### 5.1.4 Behavioral Data Faceted Violin Plot

```
ggplot(BehavDat, aes(Variable, OR, color=Behavior))+
  geom_violin(trim=FALSE)+
  geom_hline(yintercept = 1)+
  labs(x="Activity & Behavior Stressors", y="Odds Ratio (OR)")+
  stat_summary(fun.data=mean_se, geom="pointrange")+
  facet_grid(~Behavior)+
  coord_flip()+
  theme_bw(14)+theme(text=element_text(size=14, family="serif"))+
  theme(legend.position="none", strip.text = element_text( size=14, colour="black"),
        strip.background = element_rect(fill="white", colour="black",size=1))
```

### 5.1.5 Social Data Faceted Violin Plot

```
ggplot(SocialDat, aes(Variable, OR, color=Social))+
  geom_violin(trim=FALSE)+
  geom_hline(yintercept = 1)+
  labs(x="Social Stressors", y="Odds Ratio (OR)")+
  stat_summary(fun.data=mean_se, geom="pointrange")+
  facet_grid(~Social)+
  coord_flip()+
  theme_bw(14)+theme(text=element_text(size=14, family="serif"))+
  theme(legend.position="none", strip.text = element_text( size=14, colour="black"),
        strip.background = element_rect(fill="white", colour="black",size=1))
```

### 5.1.6 Chemical Data Faceted Violin Plot

```
ggplot(ChemicalDat, aes(Class, OR, color=Chemical))+
  geom_violin(trim=FALSE)+
  geom_hline(yintercept = 1)+
  labs(x="Chemical Stressors", y="Odds Ratio (OR)")+
  stat_summary(fun.data=mean_se, geom="pointrange")+
  facet_grid(~Chemical)+
  coord_flip()+
  theme_bw(14)+theme(text=element_text(size=14, family="serif"))+
  theme(legend.position="none", strip.text = element_text( size=14, colour="black"),
        strip.background = element_rect(fill="white", colour="black",size=1))
```

---

# 6 Senstivity Analysis

## 6.1 Conduct Senstivity Analysis with the *metafor* package.

Use the meta-analysis code with all estimators for comparison.

### 6.1.1 Maternal Data Code:

```
rma(OR, V, data=AllCogDat, subset=(Factors=="Maternal"), method="FE")
rma(OR, V, data=AllCogDat, subset=(Factors=="Maternal"), method="HE")
rma(OR, V, data=AllCogDat, subset=(Factors=="Maternal"), method="DL")
rma(OR, V, data=AllCogDat, subset=(Factors=="Maternal"), method="PM")
rma(OR, V, data=AllCogDat, subset=(Factors=="Maternal"), method="EB")
rma(OR, V, data=AllCogDat, subset=(Factors=="Maternal"), method="ML")
```

### 6.1.2 Inherent Data Code:

```
rma(OR, V, data=AllCogDat, subset=(Factors=="Inherent"), method="FE")
rma(OR, V, data=AllCogDat, subset=(Factors=="Inherent"), method="HE")
rma(OR, V, data=AllCogDat, subset=(Factors=="Inherent"), method="DL")
rma(OR, V, data=AllCogDat, subset=(Factors=="Inherent"), method="PM")
rma(OR, V, data=AllCogDat, subset=(Factors=="Inherent"), method="EB")
rma(OR, V, data=AllCogDat, subset=(Factors=="Inherent"), method="ML")
```

### 6.1.3 Behavioral Data Code:

```
rma(OR, V, data=AllCogDat, subset=(Factors=="Behavioral"), method="FE")
rma(OR, V, data=AllCogDat, subset=(Factors=="Behavioral"), method="HE")
rma(OR, V, data=AllCogDat, subset=(Factors=="Behavioral"), method="DL")
rma(OR, V, data=AllCogDat, subset=(Factors=="Behavioral"), method="PM")
rma(OR, V, data=AllCogDat, subset=(Factors=="Behavioral"), method="EB")
rma(OR, V, data=AllCogDat, subset=(Factors=="Behavioral"), method="ML")
```

### 6.1.4 Social Data Code:

```
rma(OR, V, data=AllCogDat, subset=(Factors=="Social"), method="FE")
rma(OR, V, data=AllCogDat, subset=(Factor=="Social"), method="HE")
rma(OR, V, data=AllCogDat, subset=(Factor=="Social"), method="DL")
rma(OR, V, data=AllCogDat, subset=(Factor=="Social"), method="PM")
rma(OR, V, data=AllCogDat, subset=(Factor=="Social"), method="EB")
rma(OR, V, data=AllCogDat, subset=(Factor=="Social"), method="ML")
```

### 6.1.5 Chemical Data Code:

```
rma(OR, V, data=AllCogDat, subset=(Factor=="Chemical"), method="FE")
rma(OR, V, data=AllCogDat, subset=(Factor=="Chemical"), method="HE")
rma(OR, V, data=AllCogDat, subset=(Factor=="Chemical"), method="DL")
rma(OR, V, data=AllCogDat, subset=(Factor=="Chemical"), method="PM")
rma(OR, V, data=AllCogDat, subset=(Factor=="Chemical"), method="EB")
rma(OR, V, data=AllCogDat, subset=(Factor=="Chemical"), method="ML")
```

---

# 7 Pubilcation Bias

## 7.1 Test for Pubilcation Bias with the *metafor* package.

Use both SE and 1/n as the predictor for comparison [see description in text].

### 7.1.1 Maternal Data Code:

```
regtest.default(MaternalDat$OR, MaternalDat$V, MaternalDat$SE,MaternalDat$ni, 
                predictor = "se", "ni" = MaternalDat$n, 
                digit= 2, verbose = TRUE, control = list(stepadj=0.5))
regtest.default(MaternalDat$OR, MaternalDat$V, MaternalDat$SE, MaternalDat$ni, 
                predictor = "ni", "ni" = (1/MaternalDat$n), digit= 2, 
                verbose = TRUE, control = list(stepadj=0.5))
```

### 7.1.2 Inherent Data Code:

```
regtest.default(InherentDat$OR, InherentDat$V, InherentDat$SE,InherentDat$ni, 
                predictor = "se", "ni" = InherentDat$n, digit= 2, 
                verbose = TRUE, control = list(stepadj=0.5))
regtest.default(InherentDat$OR, InherentDat$V, InherentDat$SE, InherentDat$ni, 
                predictor = "ni", "ni" = (1/InherentDat$n), digit= 2, 
                verbose = TRUE, control = list(stepadj=0.5))
```

### 7.1.3 Behavioral Data Code:

```
regtest.default(BehavDat$OR, BehavDat$V, BehavDat$SE, BehavDat$ni, 
                predictor = "se", "ni" = BehavDat$n, digit= 2, 
                verbose = TRUE, control = list(stepadj=0.5))
regtest.default(BehavDat$OR, BehavDat$V, BehavDat$SE, BehavDat$ni, 
                predictor = "ni", "ni" = (1/BehavDat$n), digit= 2, 
                verbose = TRUE, control = list(stepadj=0.5))
```

### 7.1.4 Social Data Code:

```
regtest.default(SocialDat$OR, SocialDat$V, SocialDat$SE, SocialDat$ni, 
                predictor = "se", "ni" = SocialDat$n, digit= 2, 
                verbose = TRUE, control = list(stepadj=0.5))
regtest.default(SocialDat$OR, SocialDat$V, SocialDat$SE,SocialDat$ni, 
                predictor = "ni", "ni" = (1/SocialDat$n), digit= 2, 
                verbose = TRUE, control = list(stepadj=0.5))
```

### 7.1.5 Chemical Data Code:

```
regtest.default(ChemicalDat$OR, ChemicalDat$V, ChemicalDat$SE, ChemicalDat$ni, 
                predictor = "se", "ni" =ChemicalDat$n, digit= 2, 
                verbose = TRUE, control = list(stepadj=0.5))
regtest.default(ChemicalDat$OR, ChemicalDat$V, ChemicalDat$SE, ChemicalDat$ni, 
                predictor = "ni", "ni" = (1/ChemicalDat$n), digit= 2, 
                verbose = TRUE, control = list(stepadj=0.5))
```

---
