# Supplementary material for: A Meta-Analysis of Stressors from the Total Environment Associated with Children’s General Cognitive Ability
Source: Int J Environ Res Public Health. 2020 Jul 29;17(15):5451. doi: 10.3390/ijerph17155451 (PMC7432904; doi:10.3390/ijerph17155451)
Supplement: Supplementary file 1 [file ijerph-17-05451-s001.zip › Nilsen et al supplenmatry text.docx]

**Formatted for:** International Journal of Environmental Research and Public Health

A Meta-Analysis of Stressors from the Total Environment Associated with Children’s General Cognitive Ability

**Supplemental Text**

Frances M. Nilsen^1,2,*^, Jazmin D.C. Ruiz^1,3^ and Nicolle S. Tulve^2^

^1^Oak Ridge Institute for Science and Education Post-Doctoral Participant stationed at U.S. Environmental Protection Agency, Office of Research and Development, Research Triangle Park, NC; [Nilsen.frances@epa.gov](mailto:Nilsen.frances@epa.gov); 919-541-2574

^2^U.S. Environmental Protection Agency, Office of Research and Development, Research Triangle Park, NC; tulve.nicolle@epa.gov

^3^Honeywell International Inc., Buffalo, NY; jazmin.ruiz@honeywell.com

*author to whom all correspondence should be addressed

3.1. Prenatal Exposures

3.1.1. Maternal Factors

Substance Use

When all substance use stressors were examined together, we observed a statistically significant relationship that had a 3% decreased likelihood to affect childhood cognitive ability (OR = 0.97, *p* ≤ 0.001, Table 1, Figure 1). When each substance use stressor was analyzed separately, all individual relationships were statistically significant, but none of the variables were observed to have an increased impact on childhood cognitive ability (Table 1).
 The individual analysis revealed that the amount of alcohol consumed had a higher OR (OR = 0.99) than binging alcohol, smoking cigarettes, (both ORs = 0.96, *p* values ≤ 0.001), using cocaine (OR = 0.96, *p* ≤ 0.01), and other narcotics (OR = 0.93, *p* ≤ 0.001; Table 1). The higher OR suggests that all other variables have less of an influence on childhood cognitive outcomes than the amount of alcohol a mother consumes, and even the amount of alcohol is not related to an increase in effect on cognition.

3.1.2. Inherent Characteristics

Child Health

The child health information included child medical history, iron deficiency, and genetic disorders. When all variables were examined together, child health was observed to have no effect on childhood cognitive ability (OR = 1.0, *p* ≤ 0.001, Table 1, Figure 2). When medical history was examined individually, it was observed to have a 3% increased effect on childhood cognitive ability (OR = 1.03, *p* ≤ 0.001). The original studies included in the medical history variable group examined significant health issues, specifically: frequency of illness, time in neonatal intensive care unit, and if/number of times resuscitation occurred [1-6]. The increased influence on cognitive ability could be due to the long-term implications of each health issue and the diversity of issues included in the analysis may be why the effect is 3% and not higher. Both iron deficiency (OR = 0.89, *p* ≤ 0.001) and genetic disorders (OR = 0.99, *p* ≤ 0.001) were observed to have a decreasing impact on cognitive development and so may be of less concern to childhood cognitive development than other examined variables.

3.2. Childhood Stressors

3.2.2. Activities and behaviors

Sleep

The stressors included in the sleep group were sleep duration, snoring behavior, and sleep-disordered breathing. When all were examined together in the RMA, sleep stressors were observed to have a significant decrease on the likelihood of effect on childhood cognitive ability (OR = 0.82, *p* ≤ 0.001, Table 1, Figure 5). When the variables were analyzed separately, only sleep duration increased its impact on childhood cognitive ability (OR = 1.06, *p* ≤ 0.01). Sleep-disordered breathing had a significant likelihood of a decreased impact on cognition (OR = 0.77, *p* ≤ 0.01), and snoring behavior (OR = 0.66, *not significant*), did not have a significant relationship to childhood cognitive ability. Recent studies have shown a relationship between sleep-wake patterns and cognitive development in infants, but there does not appear to be enough data to support a conclusive causative relationship [7, 8]. Ednick, et al. [7] suggest longitudinal studies to follow cohorts throughout different lifestages to determine if a causative relationship can be determined.

3.2.3. Social Factors

Home/Family Lifestyle

The home/family lifestyle stressors were observed to have a significant 2% decrease in their likelihood of affecting childhood cognitive ability when they were combined in the RMA (OR = 0.98. *p* ≤ 0.001, Table 1, Figure 4). Individual variables included in the home/family lifestyle group were observed to have similar results. Number of siblings (OR = 0.94, *p* ≤ 0.01), caregiver relationship (OR = 0.92, *p* ≤ 0.01), and home location (OR = 0.99, *p* ≤ 0.001) were all observed to have a significant decrease in their likelihood of influence on cognition, ranging from 1% – 8%. Marital status was observed to have a significant (1%) increase in likelihood of effect (OR = 1.01, *p* ≤ 0.001). Surprisingly, the collective home/family lifestyle stressors (e.g., marital status) did not have an increased influence on childhood cognitive ability. Previous research has shown that marital status, home environment parental responsivity, and the availability of stimulating play materials are all related to increased childhood IQ/positive developmental outcomes and that siblings affect cognition more than peers [9-11]. However, the home/family lifestyle stressors evaluated in our analysis appear to be of less influence than marital status on childhood cognitive ability.

**Supplemental References**

1. Odd, D. E.; Lewis, G.; Whitelaw, A.; Gunnell, D., Resuscitation at birth and cognition at 8 years of age: a cohort study. *Lancet* **2009,** 373, (9675), 1615-22.

2. Carlo, W. A.; Goudar, S. S.; Pasha, O.; Chomba, E.; McClure, E. M.; Biasini, F. J.; Wallander, J. L.; Thorsten, V.; Chakraborty, H.; Wright, L. L., Neurodevelopmental outcomes in infants requiring resuscitation in developing countries. *The Journal of pediatrics* **2012,** 160, (5), 781-5.e1.

3. Schermann, L.; Sedin, G., Cognitive function at 10 years of age in children who have required neonatal intensive care. *Acta paediatrica (Oslo, Norway : 1992)* **2004,** 93, (12), 1619-29.

4. Koutra, K.; Chatzi, L.; Roumeliotaki, T.; Vassilaki, M.; Giannakopoulou, E.; Batsos, C.; Koutis, A.; Kogevinas, M., Socio-demographic determinants of infant neurodevelopment at 18 months of age: Mother-Child Cohort (Rhea Study) in Crete, Greece. *Infant behavior & development* **2012,** 35, (1), 48-59.

5. Emond, A. M.; Blair, P. S.; Emmett, P. M.; Drewett, R. F., Weight faltering in infancy and IQ levels at 8 years in the Avon Longitudinal Study of Parents and Children. *Pediatrics* **2007,** 120, (4), e1051-8.

6. Cabrera, N. J.; Fagan, J.; Wight, V.; Schadler, C., Influence of mother, father, and child risk on parenting and children's cognitive and social behaviors. *Child development* **2011,** 82, (6), 1985-2005.

7. Ednick, M.; Cohen, A. P.; McPhail, G. L.; Beebe, D.; Simakajornboon, N.; Amin, R. S., A review of the effects of sleep during the first year of life on cognitive, psychomotor, and temperament development. *Sleep* **2009,** 32, (11), 1449-1458.

8. Gertner, S.; Greenbaum, C. W.; Sadeh, A.; Dolfin, Z.; Sirota, L.; Ben-Nun, Y., Sleep–wake patterns in preterm infants and 6 month's home environment: implications for early cognitive development. *Early human development* **2002,** 68, (2), 93-102.

9. Bradley, R. H.; Caldwell, B. M.; Rock, S. L.; Ramey, C. T.; Barnard, K. E.; Gray, C.; Hammond, M. A.; Mitchell, S.; Gottfried, A. W.; Siegel, L., Home environment and cognitive development in the first 3 years of life: A collaborative study involving six sites and three ethnic groups in North America. *Developmental psychology* **1989,** 25, (2), 217.

10. Azmitia, M.; Hesser, J., Why siblings are important agents of cognitive development: A comparison of siblings and peers. *Child development* **1993,** 64, (2), 430-444.

11. Bacharach, V. R.; Baumeister, A. A., Effects of maternal intelligence, marital status, income, and home environment on cognitive development of low birthweight infants. *Journal of Pediatric Psychology* **1998,** 23, (3), 197-205.
